# Supplementary material for: Genomic analysis of carbapenemase-encoding plasmids and antibiotic resistance in carbapenem-resistant Klebsiella pneumoniae isolates from Vietnam, 2021
Source: Microbiol Spectr. 2025 Apr 15;13(5):e03115-24. doi: 10.1128/spectrum.03115-24 (PMC12054150; doi:10.1128/spectrum.03115-24)

Supplementary Material

Genomic analysis of carbapenemase-encoding plasmids and antibiotic resistance in carbapenem-resistant *Klebsiella pneumoniae* isolates from Vietnam, 2021

Table of contents

Supplementary methods *2*

Supplementary dataset (separate Excel file). Overview of sequencing data analysis, including plasmids, antibiotic resistance, gene mutations, virulence and sequencing statistics. *3*

Supplementary figure 1. Genomic content of the (a) blaNDM-4 and (b) blaNDM-1 and blaNDM-5 carrying plasmids. *4*

Supplementary figure 2. Comparison of the different variants of bla$_{NDM-4}$-carrying plasmids found in this study. *5*

Supplementary figure 3. Comparison of the different variants of bla$_{NDM-1}$-carrying plasmids found in this study. *6*

Supplementary figure 4. Genomic content of the (a) bla$_{OXA-48}$ and bla$_{OXA-181}$ and (b) bla$_{KPC-2}$ carrying plasmids. *7*

Supplementary figure 5. Comparison of the different variants of bla$_{OXA-48}$-carrying plasmids found in this study. *8*

Supplementary figure 6. Comparison of the different variants of bla$_{KPC-2}$-carrying plasmids found in this study. *9*

Supplementary methods

Phenotypic confirmation of hypervirulence

Since hypermucoviscosity refers to the overproduction of capsular polysaccharides by hypervirulent *K. pneumoniae* and is associated with increased viscosity, this virulence trait was assessed by sedimentation assay. Briefly, bacterial cultures were incubated shaken at 37 °C for 24 h and centrifuged (1,000 x *g*, 5 min, room temperature). Hypermucoviscosity was expressed as the ratio of supernatant to total OD_{600}. Quantitative analysis of siderophore secretion was performed using the SideroTec-Total kit (Accuplex Diagnostics, Kildare, Ireland) according to the manufacturer's instructions. To limit iron(III) supply and thereby induce siderophore secretion, the bacterial cultures were incubated shaken for 24 h at 37 °C in chelated M9 minimal medium supplemented with casamino acids (c-M9-CA). The c-M9-CA consisted of the following components: M9 minimal salt medium (MP Biomedicals, Irvine, CA, USA), 2 mM $MgSO_4$ (Carl Roth, Karlsruhe, Germany), 200 µM 2,2′-dipyridyl (Carl Roth, Karlsruhe, Germany), and 0.3% (w/v) casamino acids (BD, Franklin Lakes, NJ, USA). Siderophore secretion was expressed as siderophore concentration in the supernatant.

Serum resistance was expressed as the $\log_2$ fold change in colony forming units per mL (CFU/mL) after incubation in 50% complement-containing human serum in relation to inoculum size. Percent survival in 50 mg/mL bile salts (an equal mixture of cholic acid and deoxycholic acid) was determined by assessing the differences in CFU/mL after 4 h of incubation with respect to the inoculum count. Infection of *G. mellonella* larvae was conducted in accordance with a standardised protocol. In brief, bacterial suspensions were adjusted to 10^7 CFU/mL in phosphate-buffered saline (PBS; Thermo Fisher Scientific, Waltham, MA, USA). Larvae (Deichgrille, Neumünster, Germany) were randomly divided into groups of 10 individuals each and 10 µL of adjusted bacterial suspensions were injected into the left proleg. In addition, 10 µL of PBS was injected into one group of larvae to ensure that death was not due to trauma from the injection. Each group was placed in 90 mm Petri dishes, kept at 37 °C in the dark, and death was recorded every 12 h for 48 h and at 72 h post-infection. Individuals were considered dead when they were unresponsive to physical stimuli and showed pigmentation. The results of three independent experiments were pooled for each strain to generate Kaplan-Meier plots of survival rates.

Supplementary dataset (separate excel file). Overview of sequencing data analysis, including plasmids, antibiotic resistance, gene mutations, virulence and sequencing statistics.

Supplementary figure 1. Genomic content of the (a) blaNDM-4 and (b) blaNDM-1 and blaNDM-5 carrying plasmids. Complete plasmids were compared using Average Nucleotide Identity using ANIclustermap (v.1.1.0) and representative sequences of the resulting clusters (ANI ≥ 99.99%) were visualized using R package gggenomes.

a

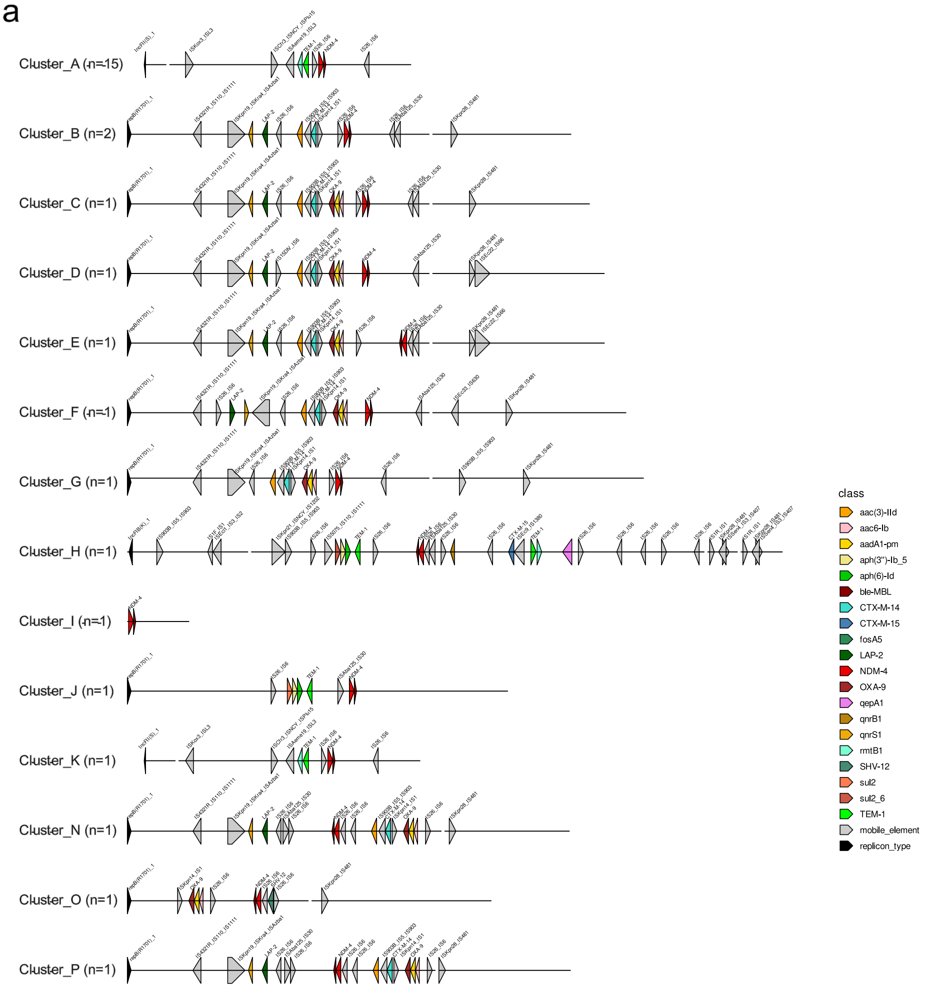

b

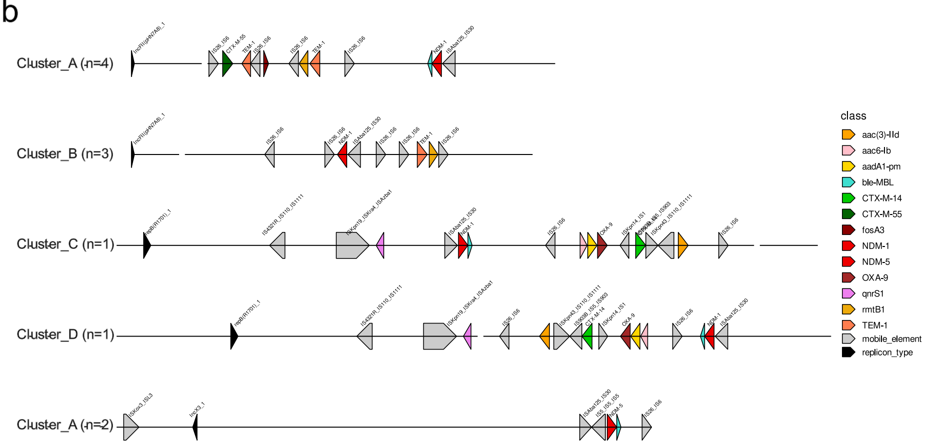

Supplementary figure 2. Comparison of the different variants of *bla*$_{NDM-4}$-carrying plasmids found in this study.
Coloured arrows are used to indicate similar genes, with links drawn between similar genes on neighbouring clusters and shaded based on sequence identity.

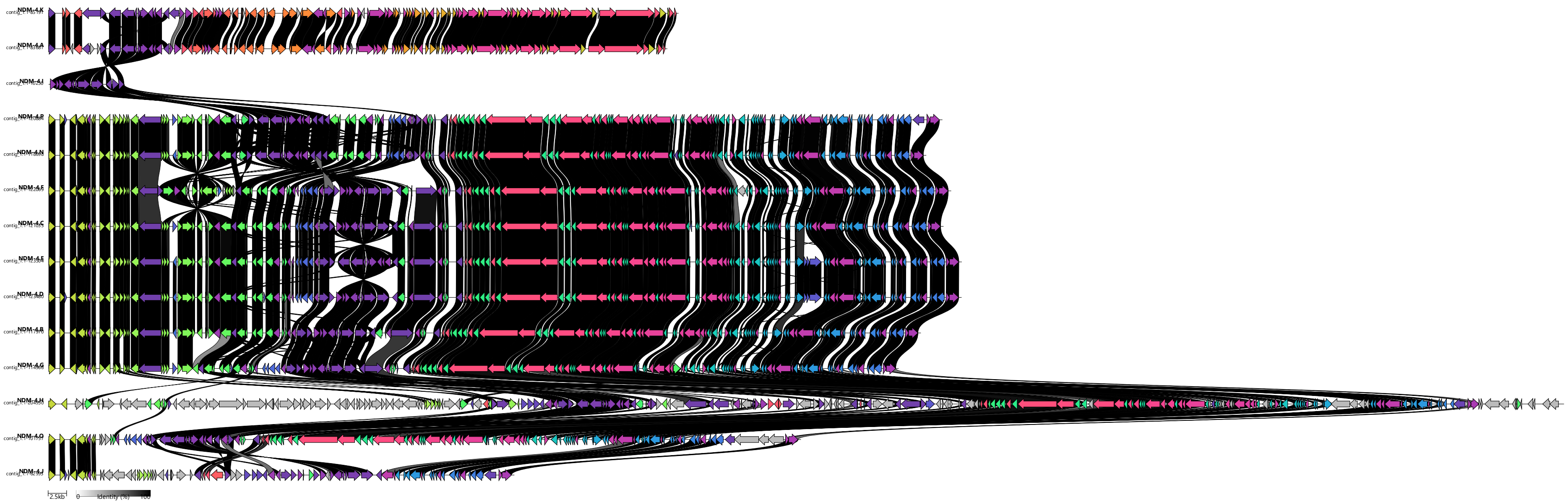

Supplementary figure 3. Comparison of the different variants of *bla*$_{\text{NDM-1}}$-carrying plasmids found in this study.
Colored arrows are used to indicate similar genes, with links drawn between similar genes on neighbouring clusters and shaded based on sequence identity.

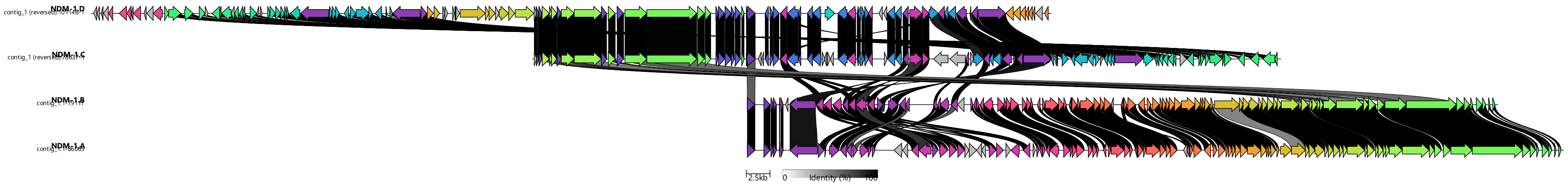

Supplementary figure 4. Genomic content of the (a) $\textit{bla}_{\text{OXA-48}}$ and $\textit{bla}_{\text{OXA-181}}$ and (b) $\textit{bla}_{\text{KPC-2}}$ carrying plasmids. Complete plasmids were compared using Average Nucleotide Identity using ANIclustermap (v.1.1.0) and representative sequences of the resulting clusters (ANI ≥ 99.99%) were visualized using R package gggenomes.

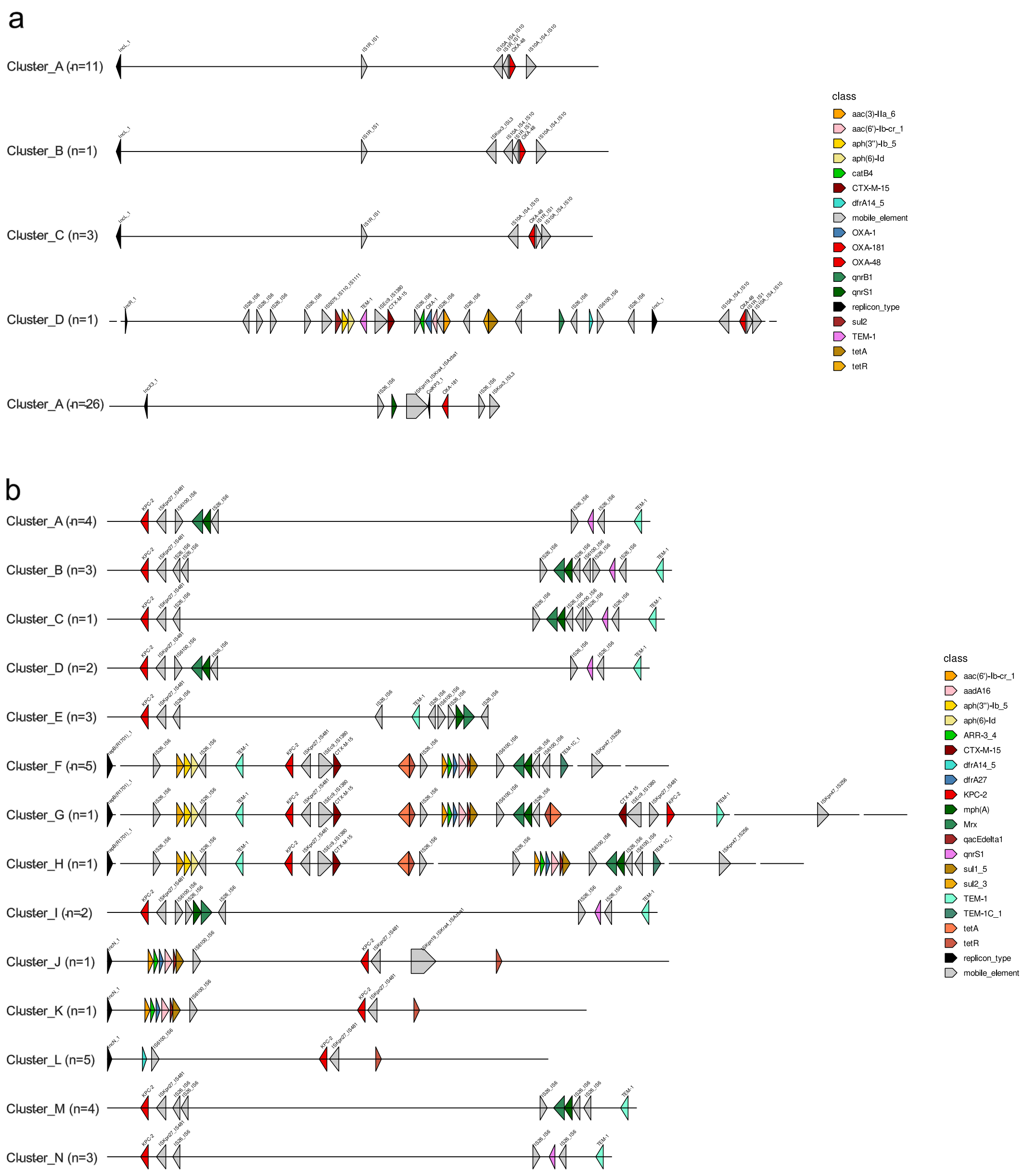

Supplementary figure 5. Comparison of the different variants of *bla*$_{\mathrm{OXA\text{-}48}}$-carrying plasmids found in this study.
Coloured arrows are used to indicate similar genes, with links drawn between similar genes on neighbouring clusters and shaded based on sequence identity.

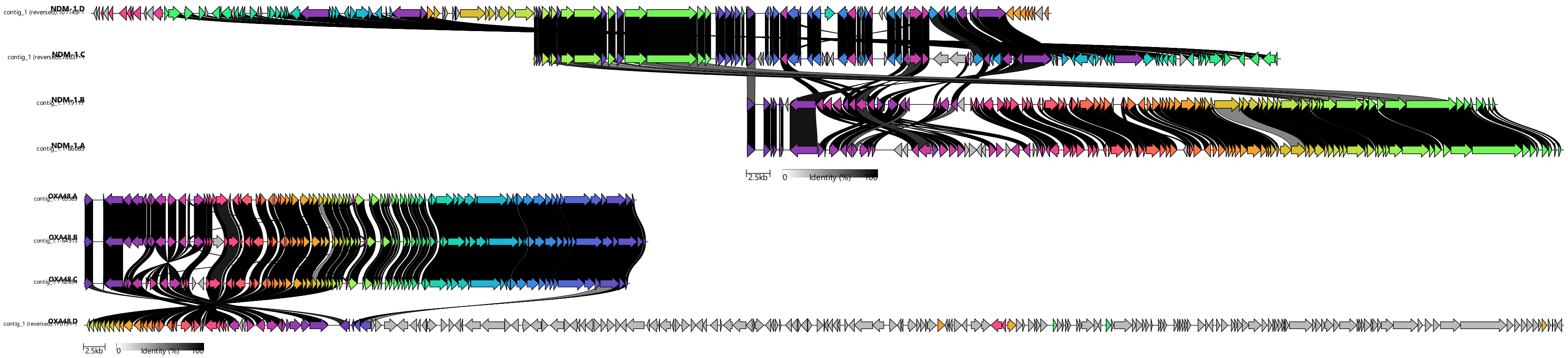

Supplementary figure 6. Comparison of the different variants of *bla*$_{KPC-2}$-carrying plasmids found in this study.

Coloured arrows are used to indicate similar genes, with links drawn between similar genes on neighbouring clusters and shaded based on sequence identity.

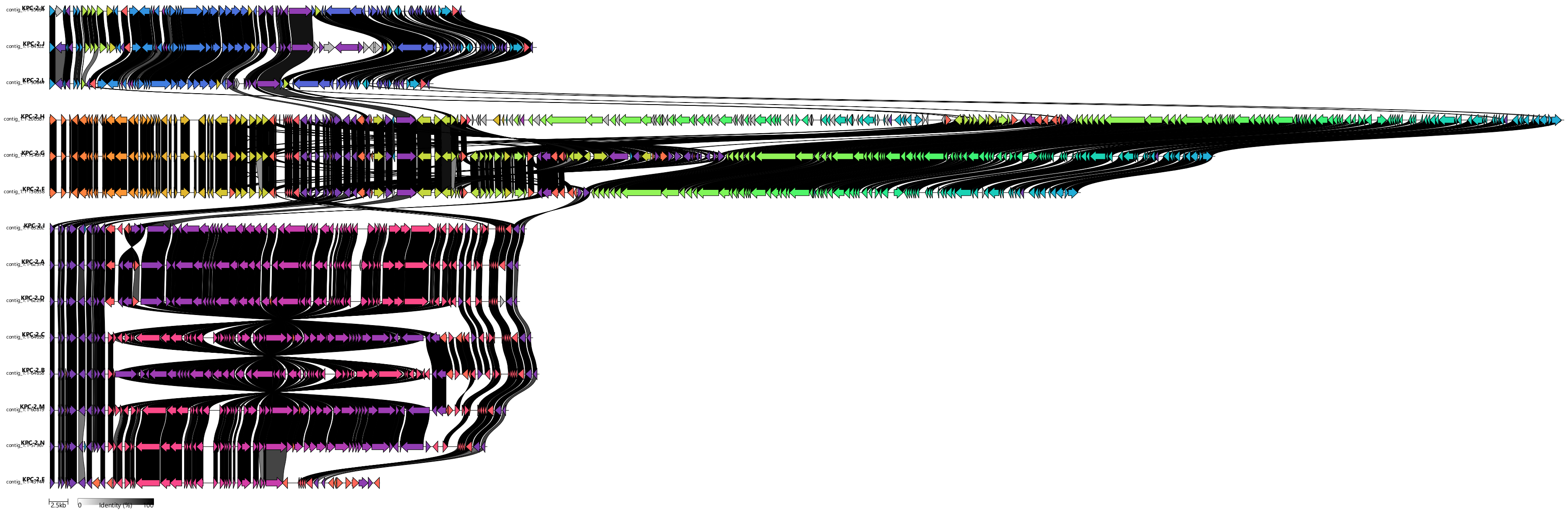

Supplement: Supplemental material — Supplemental Methods; Fig. S1 to S6. [file spectrum.03115-24-s0002.pdf]
